# Intra-atrial Course of the Right Coronary Artery: A Report of a Rare Case

**DOI:** 10.7759/cureus.100437

**Published:** 2025-12-30

**Authors:** Dhanush Tavva, Stephen Manning, Akshaya S Kalavakunta, Jose Ricardo Po, Jagadeesh K Kalavakunta

**Affiliations:** 1 College of Osteopathic Medicine, Michigan State University, Lansing, USA; 2 Cardiology, Beacon Kalamazoo Hospital, Kalamazoo, USA

**Keywords:** catheterization risk, chest pain, coronary anomaly, intra-atrial course, mdcta, right coronary artery

## Abstract

Coronary artery anomalies (CAAs) are congenital variations involving the origin, course, or termination of coronary arteries. Among these, an intra-atrial course of the right coronary artery (RCA) is a rare anatomical variant with significant clinical implications, particularly during interventional or surgical procedures. We report the case of a 69-year-old man who presented with chest discomfort and was diagnosed with an intra-atrial RCA using multi-detector computed tomography angiography (MDCTA). Although typically asymptomatic, this anomaly warrants recognition to avoid inadvertent injury during cardiac interventions such as catheterization, pacemaker implantation, or surgery. MDCTA revealed no significant coronary artery stenosis, and the patient was managed conservatively without invasive coronary angiography.

## Introduction

Coronary artery anomalies (CAAs) are rare congenital deviations in the origin, course, or termination of coronary arteries. One such anomaly is the intra-atrial course of the right coronary artery (RCA), in which a segment of the artery traverses the right atrial cavity. The reported prevalence is approximately 0.09-0.1%, but with the advent of advanced imaging modalities such as multi-detector computed tomography angiography (MDCTA), detection rates have increased [1]. While often asymptomatic, this anomaly poses a significant risk during cardiac interventions or surgeries [2]. We present a case of a 69-year-old man with an incidentally discovered intra-atrial RCA, diagnosed non-invasively via MDCTA.

## Case presentation

A 69-year-old Caucasian man with a history of hypertension, hyperlipidemia, and mild obesity (BMI 33 kg/m²) presented with acute-onset, left-sided chest pain of three hours duration. The pain was non-pleuritic, non-exertional, and unaccompanied by nausea, vomiting, diaphoresis, or radiation. He denied shortness of breath, orthopnea, paroxysmal nocturnal dyspnea, palpitations, dizziness, edema, or recent trauma. There was no significant past medical or family history.

**Figure 1:**
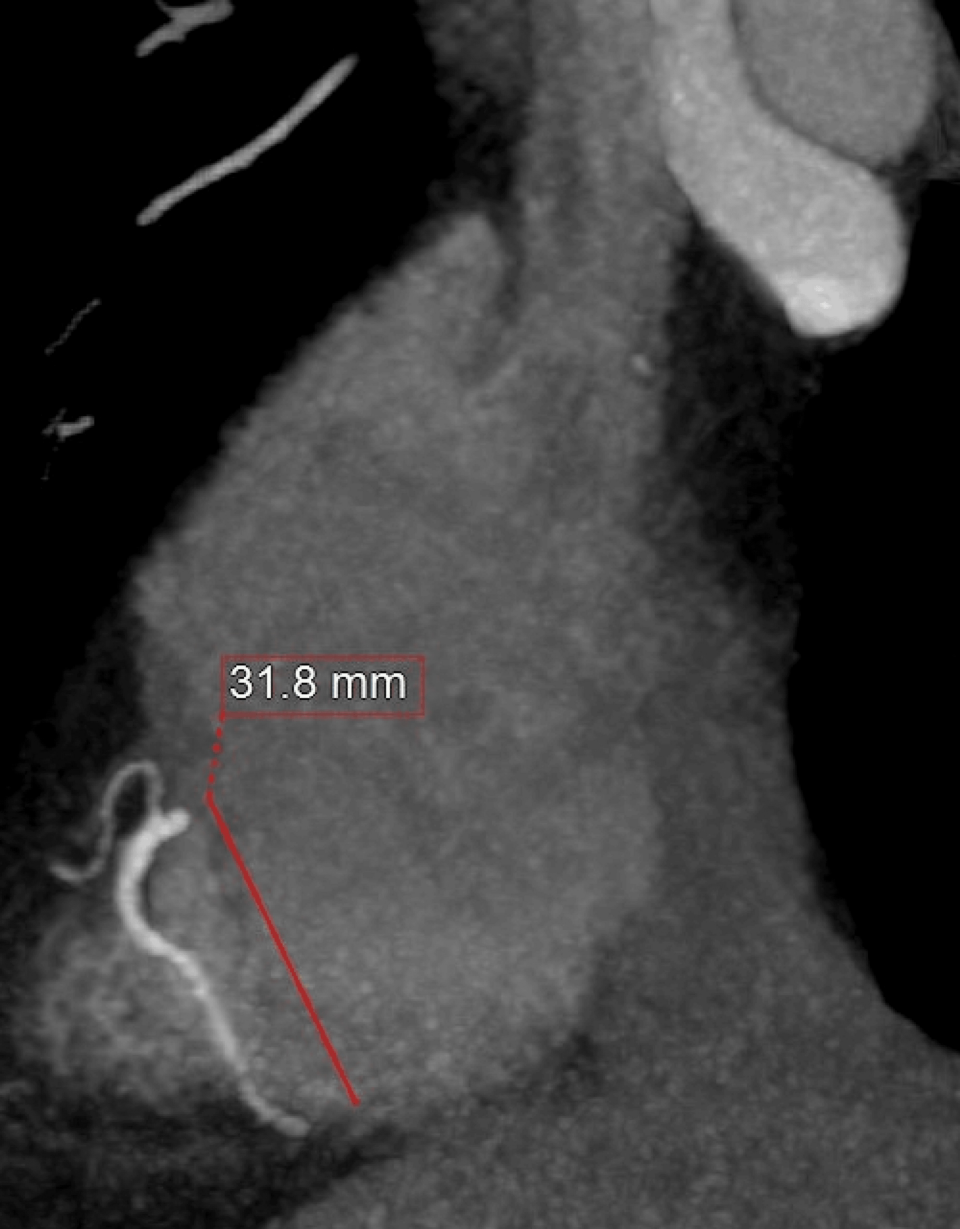
Multi-detector computed tomography angiography (MDCTA) showing the intra-atrial segment of the RCA (31.8 mm) The artery traverses the right atrium before resuming its epicardial course. MDCTA: multi-detector computed tomography angiography, RCA: right coronary artery

Vital signs were stable: blood pressure 150/85 mmHg and heart rate 78 bpm. Physical examination revealed normal heart sounds without murmurs. Laboratory tests, including troponin, creatine kinase, and D-dimer, were within normal limits. The electrocardiogram (ECG) showed a normal sinus rhythm without ST-segment changes. Given his low to intermediate risk and non-classical symptoms, the patient underwent non-invasive coronary imaging via MDCTA. The scan showed no significant coronary artery stenosis, although mild calcification was noted in the left anterior descending artery (LAD). Notably, the mid-RCA followed an anomalous intra-atrial path, penetrating the anterior wall of the right atrium before resuming its epicardial trajectory. The intra-atrial segment measured approximately 31.8 mm in length (Figure 1).

**Figure 2:**
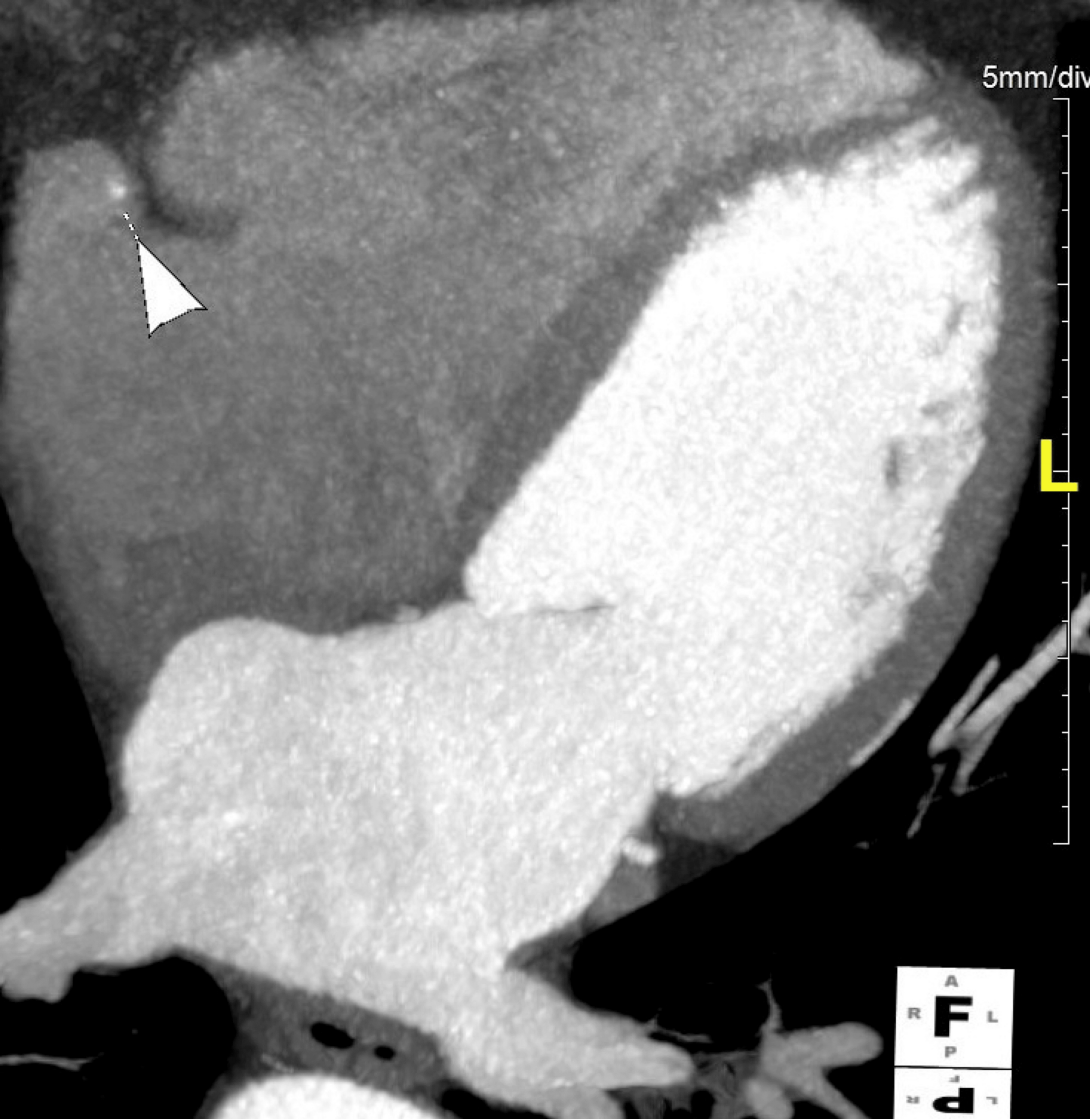
Axial MDCTA image Axial multi-detector computed tomography angiography (MDCTA) image demonstrating the intra-atrial segment of the right coronary artery (RCA), indicated by the arrowhead. The vessel is seen coursing within the right atrium, fully surrounded by intracavitary blood, confirming the anomaly.

Complete circumferential enhancement by right atrial contrast confirmed the diagnosis (Figure 2). 

**Figure 3:**
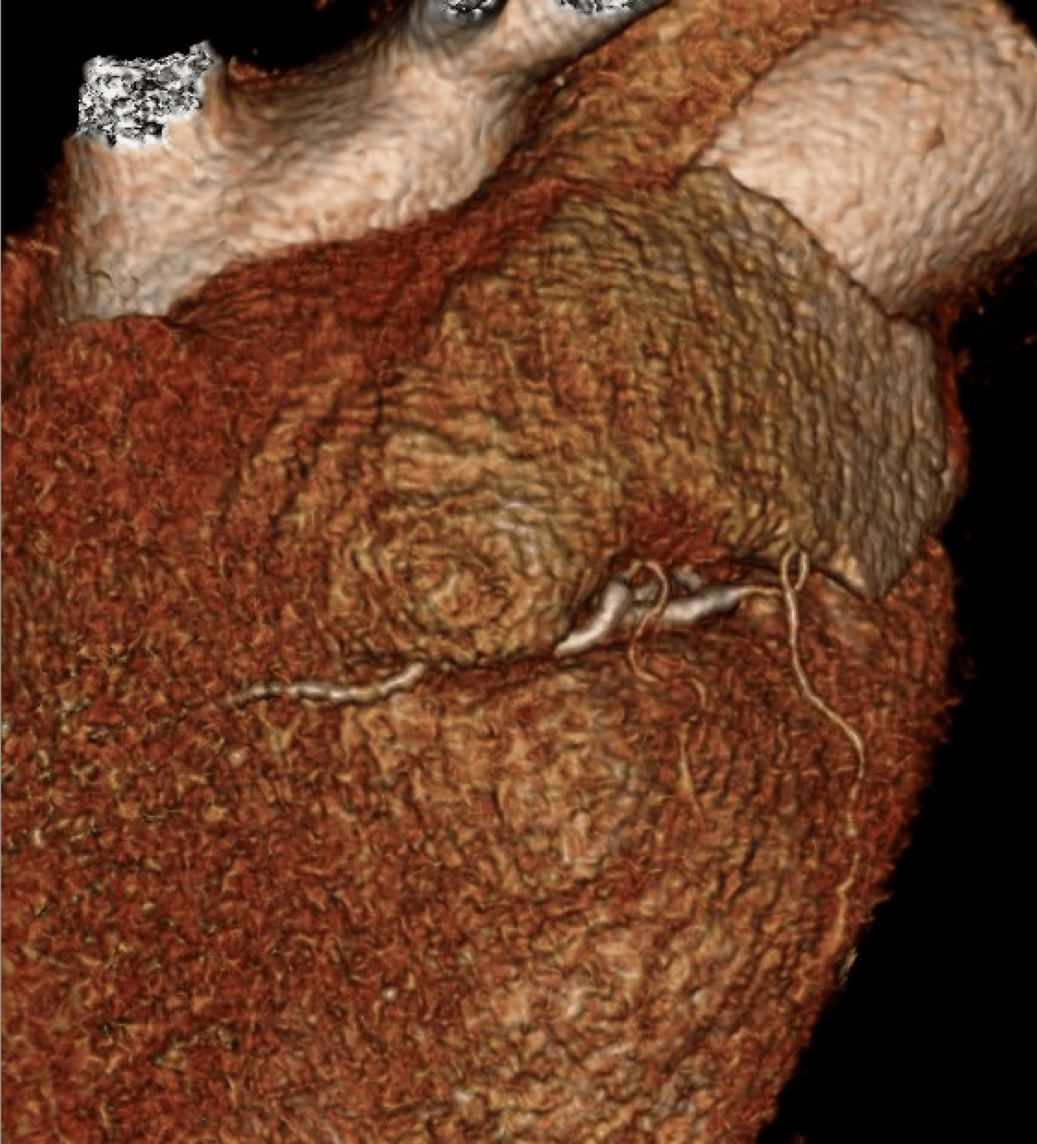
3D MDCTA reconstruction image Volume-rendered 3D multi-detector computed tomography angiography (MDCTA) reconstruction showing the anomalous intra-atrial course of the right coronary artery (RCA). The image provides clear external visualization of the vessel’s path through the right atrial wall, supporting anatomical assessment and pre-procedural planning.

The rest of the coronary anatomy was unremarkable. Multiplanar reconstruction and volume rendering clarified the vessel’s course (Figure 3).

The patient was initiated on optimal medical therapy for primary prevention of coronary artery disease: amlodipine 5 mg daily for hypertension and rosuvastatin 5 mg daily for dyslipidemia. Given the absence of obstructive lesions, invasive coronary angiography was deferred. His chest pain resolved within days, and he remained symptom-free at six-week and six-month follow-ups.

## Discussion

In the normal course, the RCA originates from the right coronary sinus with a forward and rightward takeoff, coursing exclusively along the epicardial surface before passing between the right atrial appendage and pulmonary artery into the right atrioventricular groove. It then travels within the AV groove, giving rise to conus, sinoatrial nodal, and acute marginal branches, and at the crux commonly divides into the posterior descending and posterolateral branches, reflecting a right-dominant coronary circulation in the majority of patients. The intra-atrial or intra-cavitory course of the RCA is an exceptionally rare coronary anomaly, often discovered incidentally during cardiac imaging, surgery, or autopsy [2]. In our case the diagnosis was made while evaluating for the complaint of chest pain. Its clinical significance lies in the potential for inadvertent injury during interventions involving the right atrium, such as pacemaker insertion, catheter ablation, or central line placement [1,3]. 

In our case, the anomalous trajectory of the RCA was clearly delineated using MDCTA. This imaging modality offers excellent spatial resolution and three-dimensional visualization, making it ideal for identifying coronary anomalies [4]. Recent literature suggests that improved imaging technology has increased detection rates of this anomaly to up to 1.8% [5,6]. Although usually asymptomatic, intra-atrial RCA has been associated with non-specific symptoms like chest discomfort, syncope, and palpitations. Most reported cases involve the mid-RCA, as seen in our patient, and are not associated with atherosclerosis or stenosis. One hypothesis is that the intra-atrial segment, being shielded from epicardial mechanical forces, is less prone to plaque formation. In line with earlier case reports, the intra-atrial portion of the mid-RCA in our instance measured about 31.8 mm in length. There was no evidence of severe plaque development or coronary artery stenosis. Different mechanisms were proposed for the symptoms as delineated in Table 1. 

**Table 1:** Different clinical presentations of intra-atrial right coronary artery References: [7-10] MDCTA: multidetector coronary computed tomography angiography, CT: computed tomography, ECG: electrocardiogram, PCI: percutaneous coronary intervention, EP: electrophysiology, RA: right atrium, RCA: right coronary artery, AV: atrioventricular

Presentation type	Clinical features	Proposed mechanism	Diagnostic modality	Management
Incidental / No symptoms	Asymptomatic; discovered on imaging	Benign anatomic variant	MDCTA, coronary angiography	Observation; documentation for future procedures
Chest Pain/Angina	Exertional chest pain; often normal coronaries	Dynamic kinking, altered flow during atrial systole	MDCTA; functional stress testing	Anti-anginal medical therapy^,^
Acute Coronary Syndrome (rare)	Chest pain with ischemic ECG changes or troponin elevation	Vasospasm, mechanical distortion, thrombosis	Angiography with CT confirmation	Medical therapy or PCI (case-dependent)
Arrhythmias/Palpitations	Atrial fibrillation, atrial tachycardia	Local atrial irritation or nodal ischemia	MDCTA; EP study	Medical therapy or ablation with procedural caution
Syncope / Presyncope	Exertional or unexplained syncope	Transient AV nodal or inferior wall ischemia	CT angiography; Holter monitoring	Risk stratification and tailored therapy
Procedural Complication Risk	Coronary injury during RA instrumentation	Direct RCA laceration or thrombosis	Pre-procedural MDCTA	Procedural modification or avoidance
Intraoperative finding	Identified during cardiac surgery	Aberrant coronary atrial course	Intra-operative visualization; CT	Awareness; typically no surgical correction

Accurate pre-procedural identification is critical to avoid iatrogenic RCA injury. Awareness of this anomaly should prompt cautious planning for any right atrial or adjacent cardiac procedures.

## Conclusions

The intra-atrial course of the RCA, though uncommon, carries important procedural implications. MDCTA is a valuable tool for diagnosis and pre-procedural planning. Early identification ensures safer clinical management and helps avoid potentially catastrophic complications.
